# Rubber Bandage

**Published:** 1873-05

**Authors:** U. C. Lynde


ART. IV.—*Rubber Bandage.* By U. C. LYNDE, M. D.

Compression, either by the common bandage or adhesive plaster, is employed by most surgeons in a variety of cases for the purpose of exciting the absorbents in the removal of effusions—as of blood, lymph, serum and synovia—and it has generally been recognized as a very efficient means of fulfilling the indication mentioned; but, as I usually failed with these methods of applying compression, I have been in the habit, lately, of using a bandage made of rubber tape. The fixed bandage will slacken on the least diminution of the size of the part to which it is applied. If the common bandage is applied, with a compress, over an enlarged bursa, the parts will shrink under the pressure, and, though not a drop of the contents of the bursa has been absorbed, the bandage will slacken and exert little or no influence on the absorbents. Admitting, however, that the common bandage, or adhesive plaster. will, when first applied, press upon and cause a certain amount of absorption, however little that may be, the bandage is loosened thereby, and no longer avails anything until re-applied by the surgeon; but, if the bandage is made of rubber tape, the pressure will be continuous, the diminution in the size of the part causing no appreciable difference in the pressure made by it. By means of a bandage made of this material I have been enabled to effect the cure of recently enlarged bursa over the patella and olecranon in two days, and of dropsy of the synovial membrane surrounding the tendons of the flexor sublimis and flexor profundus digitorum muscles, of six years duration, in less than a week; and that, after the failure of the common bandage, iodine, blisters, &c. The ganglions, called weeping sinews, so common about the wrist, will yield readily to this mode of bandaging.

A piece of binders' board fashioned to the size and site of the tumor, with a layer or two of cotton wadding interspersed between it and the skin, while the opposite side of the wrist is properly protected with the same material, will be all the dressings the parts will need before applying the bandage. I have in some cases bandaged the fingers and applied a wide and well padded splint to the opposite side of the wrist to avoid all possibility of strangulating the circulation in the hand. In using the rubber tape as a bandage it is only necessary to apply it over the tumor, the common roller being used to complete the dressing. Wherever compression is to be used with a view of producing absorption, there is none but the rubber bandage that is worthy of a trial; but we need an article, manufactured expressly for surgical use, that shall vary in width and power to suit emergencies, but the rubber tape that can be procured at any variety store will answer in most cases.

